# The effect of disagreement on children’s source memory performance

**DOI:** 10.1371/journal.pone.0249958

**Published:** 2021-04-09

**Authors:** Johannes B. Mahr, Olivier Mascaro, Hugo Mercier, Gergely Csibra

**Affiliations:** 1 Department of Psychology, Harvard University, Cambridge, MA, United States of America; 2 Integrative Neuroscience and Cognition Center, CNRS & Université de Paris, Paris, France; 3 Département d’études cognitives, ENS, EHESS, Institut Jean Nicod, PSL University, CNRS, Paris, France; 4 Cognitive Development Center, Department of Cognitive Science, Central European University, Budapest, Hungary; 5 Department of Psychological Sciences, Birkbeck, University of London, London, United Kingdom; Abertay University, UNITED KINGDOM

## Abstract

Source representations play a role both in the formation of individual beliefs as well as in the social transmission of such beliefs. Both of these functions suggest that source information should be particularly useful in the context of interpersonal disagreement. Three experiments with an identical design (one original study and two replications) with 3- to 4-year-old-children (N = 100) assessed whether children’s source memory performance would improve in the face of disagreement and whether such an effect interacts with different types of sources (first- vs. second-hand). In a 2 x 2 repeated-measures design, children found out about the contents of a container either by looking inside or being told (IV1). Then they were questioned about the contents of the container by an interlocutor puppet who either agreed or disagreed with their answer (IV2). We measured children’s source memory performance in response to a free recall question (DV1) followed by a forced-choice question (DV2). Four-year-olds (but not three-year-olds) performed better in response to the free recall source memory question (but not the forced-choice question) when their interlocutor had disagreed with them compared to when it had agreed with them. Children were also better at recalling ‘having been told’ than ‘having seen’. These results demonstrate that by four years of age, source memory capacities are sensitive to the communicative context of assertions and serve social functions.

## Introduction

The ability to ‘know how we know’—to know the sources of our beliefs—plays an important role in belief formation and transmission. The source of a piece of information can have a dramatic impact on its cognitive effects in belief formation: people update their beliefs on the basis of sources they view as reliable and tracking where a belief comes from allows people to adjust their trust in the source if it turns out that the belief was false (e.g., [1, 2]). Apart from playing a role in belief formation, knowing where our beliefs come from also influences how we communicate those beliefs to others. Claims can be undermined if they are linked to an inappropriate source (e.g., if someone asserts that it will rain tomorrow because they dreamt it), or bolstered if they are attributed to an authoritative source (e.g., if your doctor tells you that you are sick; see [3]).

Thus, when children expect that source information will play an important communicative role, they might be more likely to explicitly remember source information. In particular, source information will be useful in interpersonal disagreement, as one can report sources in an attempt to convince a disagreeing conversational partner (e.g., “I saw it”), or to mitigate the disagreement by hedging one’s position (e.g., “someone told me”). In order to assess whether preschoolers are sensitive to these social functions of source information, we studied whether disagreement facilitates the explicit retrieval of source information.

Before introducing our experimental design, we offer a short overview of the cognitive requirements of source representation and how these capacities might be brought to bear in communication.

### The cognitive requirements of explicit source memory

In spite of its apparent simplicity, the capacity to report the sources of our beliefs is cognitively demanding. Consider the simplest and most common sources people attribute their beliefs to, such as perception, hearsay, and inference. If you form the belief that it is going to rain tomorrow after watching a weather report, did you form the belief based on perception of the map, hearsay from the presenter, or inference from this information? Competently retrieving source information is particularly challenging because it is often not obvious what the appropriate source is in a given context and different sources can have different cognitive and conversational consequences (e.g. [4–6]).

Partly as a result of the difficulty in attributing sources to our beliefs, people seem to leave most of their beliefs without source tags when they are acquired [7]. Even when sources are taken into account in belief formation (people tend to update their beliefs on the basis of sources they view as reliable), they are not always tagged to beliefs *as sources* [8, 9]. Instead, source tagging is often performed a posteriori. To do so, dedicated source monitoring mechanisms infer sources from memories of learning events and other information [10], such as assumptions about what sources are appropriate for what beliefs (for example, that “feeling” can’t be the source of knowing the color of an object [11]).

Source recall is thus a cognitively sophisticated and demanding ability. Consequently, children start developing the capacity to accurately report the sources of their beliefs relatively late (between three and five years of age, see [12–14]) compared to other social skills such as mindreading (e.g., [15–17]). Why are source representations important enough to warrant this cognitive complexity?

### The cognitive functions of explicit source memory

Source representation might serve several functions, which can be broadly classified into two categories: individual and social functions [1]. As mentioned above, source representation might enhance individual cognition by allowing people to appropriately update their beliefs. By comparing the sources of beliefs held in memory and of novel pieces of information—in particular communicated information (see [2, 18])—people might make better decisions about how to weigh these beliefs and when to revise them. Directly or not, it is chiefly this individual function that has been the focus of the developmental literature on source memory (e.g., [13, 19, 20]).

For example, Whitcombe and Robinson (2000) [21] as well as Robinson and Whitcombe (2003) [22] investigated whether 3- to 5-year-old children would accept a contradiction from an experimenter who was either better or less well informed than themselves. They tested whether children’s ability to accurately remember the source of their own belief would help them make the right decision about what to believe when the experimenter contradicted them. The results suggested that children were sensitive to how well informed a speaker was relative to themselves in their belief updating decisions. For instance, in Whitcombe and Robinson’s tunnel game studies, the participants had to identify which one of a pair of toys differing either in color or in hardness, was hidden in a tunnel. The children and an experimenter sometimes saw the hidden toy, or they felt it. Children who had only uninformative access (e.g., felt a toy identified by color) were more likely to defer to an experimenter’s claim about the toy’s identity when the latter had informative access (saw the toy), than when she did not (felt it). However, the results did not reveal a strong link between source representation and belief revision: many children were able to update their beliefs appropriately without being able to accurately remember the source of their own beliefs.

Related evidence comes from a large literature investigating how children learn from testimony. Preschoolers have been shown to have sophisticated capacities for tracking the trustworthiness and competence of informants and to use such information for assessing the reliability of assertions in belief formation [23–25]; for recent reviews see [26, 27]. While explicit source memory has not been the focus of studies investigating how children learn from testimony, these topics are closely related. Studies of children’s capacities to evaluate the reliability of speakers, for example, suggest that children track where information comes from for long enough to revise trust when a statement turns out to be inaccurate. Moreover, toddlers and preschoolers have been shown to not only monitor the reliability of speakers but to also appropriately revise their beliefs when new information about the speaker’s reliability is revealed [28–30]. We term these functions ‘individual’ because they mainly concern the way a given individual manages their beliefs in the face of new information. In other words, these uses of source memory come into play mainly on the receiver’s side of informational exchanges.

Besides these individual functions, however, source information also has important social functions; that is, functions concerning the ‘sender’s side’ in the exchange of beliefs between individuals. Being able to remember the sources of their beliefs allows people to communicate these sources to others, thereby serving a variety of goals: one can use sources to convince (“I saw it with my own eyes”), to gain credit (“I found the solution on my own”), or on the contrary, to hedge one’s statements (“It’s only something I’ve heard”) (for a more general argument regarding the social functions of episodic memory, see [31]; see also [32–35]). For example, Köymen & Tomasello (2018) [36] have shown that 7-year-olds (and to some extent 5-year-olds) already use source information productively as justifications in the context of collaborative decision making. The importance of the social functions of source representation is also suggested by the fact that over one-quarter of the world’s languages employ obligatory grammatical markers, known as evidentials, specifying the source of the speaker’s assertion [37, 38 gives an estimate of over 50% of the world’s languages]. Even languages that do not encode evidentiality grammatically possess numerous ways of communicating the sources of a speaker’s beliefs [39].

Moreover, there are reasons to believe that children’s ability to use source information for social ends is closely related to the development of source memory. Although learning a language with evidentials does not seem to improve children’s source memory capacities [5] children’s production of accurate evidentials (arguably serving a social function of source memory) develops in parallel with their source memory [40]. Moreover, the production of accurate evidentials precedes children’s full comprehension of similar expressions (the latter arguably serving a more individual function of source memory) [41].

How does source information contribute to communicative exchanges? First, source information helps audiences calibrate their trust in whatever the speaker is asserting (e.g. [42]). By communicating their source, speakers leave it up to their audience to weigh the evidence according to their own estimate of the source’s credibility. Second, source claims are relevant to assess who is accountable for the validity of the communicated information. By claiming to have first-hand evidence, speakers make themselves directly accountable, while referring to a second-hand source allows speakers to defer such responsibility to someone else (see, e.g., [33, 43]). The capacity to know the source of one’s belief is therefore not only important to enable efficient belief revision: explicit source memory also plays a crucial role in helping speakers effectively communicate their beliefs to others.

### Disagreement and the social functions of source memory

In light of the above discussion, disagreement should facilitate both individual and social functions of source memory retrieval. First, disagreement might signal that the belief in question is less reliable than one might have thought otherwise. This might prompt a search for how the belief was initially acquired. Second, however, disagreement signals that the belief in question requires justification and this in turn might prompt the listener to search for source information in order to provide such justification. Moreover, a speaker might exert more scrutiny of a justification provided if the justification request follows a disagreement. As a result, a disagreement should lead people to not just search for justifications of the contested assertion but more specifically for *accurate* justifications. If source memory serves social functions, when facing disagreement speakers might thus be prompted to spend more cognitive resources on retrieving and reporting their source accurately than in other conversational contexts. Note that both of these effects bear on the retrieval rather than encoding stage of belief formation. While it is the case that, under some circumstances, source information is encoded when a given belief is formed (see e.g. [9]), disagreement cannot always be anticipated at belief formation.

Existing evidence shows that preschoolers are sensitive to some features of disagreement: 4-year-olds have been shown to detect when someone contradicts them [44], and to adjust their communication to convince an interlocutor who keeps disagreeing [45]. Given that the preschool years are crucial in the development of source memory [13], and that children of this age appear to be able to react appropriately to disagreement, they provide the most relevant age at which to test our hypothesis, namely, that source memory performance will be facilitated in a context of interpersonal disagreement, compared to a context of interpersonal agreement.

### The present study

In the current study, the children were made aware of the contents of a container either by looking inside the container themselves, or by being told what the contents were by the experimenter. The children then met an interlocutor (a puppet) who asked them about those contents. Once the child had said what they believed was in the container, the puppet either agreed or disagreed with the child’s answer. Finally, the puppet asked an open (“How do you know”?) followed by a closed (“Did you see it or did someone tell you?”) source memory question. We conducted three separate experiments with the same design: the original study (with 4-year-olds), a replication with the same age group, and a replication with younger children (3-year-olds).

While Whitcombe and Robinson (2000) [21] and Robinson and Whitcombe (2003) [22] investigated whether children would appropriately revise their own belief in the face of a contradiction by an interlocutor, they did not test the effects of this contradiction on source memory performance. Moreover, in contrast to previous studies, in our paradigm, the source memory question was not asked by the experimenter but by the interlocutor puppet who was engaged in a game with the child. This difference is important because children might interpret questions asked by a ‘third party’ experimenter as test questions [46], rather than as a natural request for justification which was our target here. While test questions assume that the speaker herself already knows the answer, requests for justification can be interpreted as a speaker’s genuine requests for more information that could potentially change their minds.

Our central prediction bore on the effect of agreement: We predicted that disagreement would facilitate source memory performance, making children more likely to recall the source of their beliefs when their interlocutor disagreed with them compared to when the interlocutor agreed with them.

## Methods

We conducted three experiments: Experiment 1 constituted the original study with 4-year-old children. Its results tended to confirm our hypothesis about the effect of agreement on source memory, by showing marginally significant statistical trends in the expected direction (see below). Thus, in order to confirm our results, we ran Experiment 2, which was a pre-registered replication of Experiment 1. Experiment 3 was designed to test the ontogeny of the behaviors observed in Experiments 1 and 2. It used the same procedure as Experiments 1 and 2 with a younger age group (3-year-olds). Since the three experiments were identical in terms of procedure and design, we report their methods and results together. All experiments were carried out in the same environment, and Experiments 2 and 3 were performed after Experiment 1. Moreover, Experiments 2 and 3 were carried out by different experimenters than Experiment 1. Given that this study was carried out in Hungarian, it bears mentioning that Hungarian does not have evidential markers.

### Participants

For Experiment 1, we recruited 34 4-year-old children. Two of these children had to be excluded because of experimenter error (see Coding and Analyses), so that the final sample included 32 children (age range: 48–59 months-old; mean age: 54 months; 16 females). We chose this number of participants so as to ensure that at least four children completed each counterbalancing order (see Design).

In Experiments 2 and 3, we aimed for a sample size superior or equal to that of the original study for each age group. We had a pre-registered stopping rule of 40 participants for each of our experiments which we exceeded due to a scheduling miscommunication between the research assistants running the study. Thus, we recruited 42 4-year-olds for Experiment 2 and 43 3-year-olds for Experiment 3. Children were excluded from analysis if they contributed fewer than four valid trials (Experiment 1: 0 children; Experiment 2: 6 children; Experiment 3: 6 children). Further, we had to exclude 2 children from Experiment 2 due to technical difficulties and 1 child from Experiment 3 for not fulfilling the age criterion.

Thus, Experiment 2’s final sample included 34 4-year-olds (age range: 48–59 months-old; mean age: 56; 18 females) and Experiment 3’s final sample included 34 3-year-olds (age range: 36–47 months-old; mean age: 43; 11 females).

All of our participants were recruited at the local Zoo in Budapest (Hungary) during visiting hours. The procedure and informed consent forms were reviewed and approved by the Hungarian United Ethical Review Committee for Research in Psychology.

### Design

All three experiments employed the same within-subjects design with two two-level independent variables: Agreement (interlocutors agrees/interlocutor disagrees) and Source (seeing/being told). Participants completed three familiarization trials and six test trials, which exposed them to each combination of factors at least once. Since piloting revealed that increasing the trial number to eight test trials caused children to lose focus in the last two trials, we chose to expose children to six test trials in order to maximize the amount of data points we could collect per participant. Even though this resulted in an unbalanced design on the participant level, all cells of our design received an equal number of observations across children.

The order in which Agreement and Source factors varied was counterbalanced within as well as across participants, and so was the order of sources mentioned in the closed source memory question. Moreover, whether children received an ‘agreement’ or ‘disagreement’ and a ‘seen’ or ‘told’ trial first was counterbalanced across children. All in all, we employed eight counterbalancing orders, aiming for a minimum of four participants per order in each group (for the full counterbalancing information see Tables 1 and 2 in S1 File).

Each child was asked three test questions per test trial: a contents question about the contents of the container, an open source memory question ("how do you know that there is a […] inside ?"), and a closed source memory question ("did you see or were you told that there is a […] inside?"). We included both an open and a closed test question in order to have two separate measures of children’s source memory. While the open source question was intended to assess children’s source memory through free recall, the closed source question was intended to serve as a recognition memory test for source.

### Procedure

Before the start of the experiment, the children were asked if they wanted to participate in a game and only participated in case both they and their parents agreed to do so (parents received and signed an informed consent form). Children were seated on a chair at a table which was placed in front of a curtain. A first experimenter sat next to the table, while a second experimenter was hidden behind the curtain controlling a puppet (“Zirmi” the cat) serving as the interlocutor for the child. Caregivers were allowed to stay in the room but were asked not to interfere with the procedure in any way. The whole procedure was video recorded. Detailed scripts for the familiarization and test procedures can be found in S2 File.

#### Familiarization trials

Children completed three familiarization trials introducing them to the setting of the task, their interlocutor (‘Zirmi the cat’), the different sources (finding out about the contents of a box either by looking inside or by being told), and the fact that Zirmi sometimes disagrees without necessarily being correct.

In the first familiarization trial, children were introduced to Zirmi and asked if they wanted to play a game in which they have to find out what was inside a box placed on the table in front of them. Next, Zirmi opened the box and showed its contents to the child.

In the second familiarization trial, the experimenter removed the box from view and placed a new object inside it. The experimenter then asked the child whether they wanted to have a look inside and tell them what was in the box. The children were then allowed to look into the box and answered the question. If the child gave an incorrect answer, the experimenter corrected them. After the child gave the correct answer, the experimenter asked the child the open source question (“*How did you find out what was inside*?”) and corrected them if they gave the wrong answer. Here, any reference to visual perception was deemed as a correct answer. Next, the experimenter turned away and Zirmi appeared from behind the curtain. Zirmi asked the child about what was inside before disagreeing with the child and suggesting a different, wrong answer. Finally, the child and Zirmi opened the box together to find out that the child had been correct about its contents.

The third and last familiarization trial was similar in structure to the second one. However, in this trial the experimenter told the child about the contents of the box and Zirmi agreed with the child about the contents. Here, in response to the open source question any reference to the experimenter or to testimony was treated as a correct answer.

#### Test trials

At the beginning of each test trial, only the first experimenter and the participant interacted, and the puppet (“Zirmi,” controlled by a second experimenter) was not present. The experimenter removed the object from the last trial from the box, in full view of the child. Next, she hid a new object inside the box underneath the table, so that the hiding (but not the object itself) was visible to the child. In each trial, a different object was hidden inside the container. Which object was hidden in each trial stayed consistent across children. Next, the experimenter asked the child if they wanted to know what was inside the box by saying: “*Look*, *I put something new inside*! *Do you want to know what it is*?”. The rest of the procedure varied depending on condition.

In the ‘told’ condition, the experimenter told the participant what object she had hidden by saying: “*I will tell you*! *It’s a [name of the object hidden inside the box*, *e*.*g*., *a car]*! *There is a [name of the object hidden inside the box*, *e*.*g*., *a car] inside this box*!”. Next, the experimenter asked: *“What’s inside*? *Can you tell me*?*”*. In case the child did not give the correct answer, the experimenter told her again what was inside the box until the participant gave the correct answer.

In the ‘seen’ condition, the experimenter allowed the child to look inside the box. She said: “*Do you want to look inside to find out what’s inside*?”. Next, she let the child open the box and look inside it, before asking: *“Did you see what’s inside*? *Can you tell me*?*”*. If the child answered correctly, the experimenter said: “*Wow*! *That’s exciting*!”. In case the child did not give the correct answer, the experimenter asked her to look inside again until the participant gave the correct answer.

Next, in all conditions, the experimenter turned away and Zirmi appeared from behind the curtain. The puppet asked the child what was inside the box by saying: “*Hello*! *Oh*! *There is the box again… What do you think is inside*?” (‘contents question’). Zirmi repeated the contents question until the child gave an answer. After the child had answered, Zirmi’s reaction differed across conditions. In the ‘agreement’ condition, Zirmi agreed by saying: “*You are right*! *There must be a [name of the object that she child said to be inside the box*, *e*.*g*., *a car] inside*.*”* In the ‘disagreement’ condition, Zirmi disagreed by saying: “*No*! *I don’t think there is an [name of the object that she child said to be inside the box*, *e*.*g*., *a car] inside*! *I think there is a [name of an alternative object*, *e*.*g*., *a watch] inside*!*”*. In each disagreement trial, Zirmi suggested a different alternative object. The alternative object Zirmi suggested to the child when disagreeing was predetermined for each trial and stayed consistent across children.

In all conditions, after either agreeing or disagreeing with the child, Zirmi asked the child the open source question (“*How do you know that there is a [name of the object that she child said to be inside the box*, *e*.*g*., *a car] inside*?”). If the child did not answer, Zirmi repeated this question once. If the child still did not answer, the trial was coded as an incorrect answer (see below). Regardless of the child’s answer to the open source question, Zirmi then asked the child the closed source question (“*Did you see or were you told that there is a [name of the object that she child said to be inside the box*, *e*.*g*., *a car] inside*?”). The order in which sources were mentioned in the closed source question was counterbalanced across trials within subjects. Again, if the child did not answer the closed question, Zirmi repeated the question once. In the disagreement condition, irrespective of the child’s answer, Zirmi responded: “*Hm*‥ *if you [saw/were told] then you must be right… there must be an [name of the object that she child said to be inside the box*, *e*.*g*., *a car] inside”*.

Next, in all conditions, Zirmi and the child opened the box together to check its contents. Finally, Zirmi said: *“Oh*! *I have to go…*!*”* and went away behind the curtains. Participants then proceeded to the next test trial, till the end of the experiment.

#### Coding

Responses were double-coded by coders unfamiliar with the hypotheses of the study. Disagreements between the two primary coders were resolved by a third coder (the first author; Cohen’s kappa across all studies and measures was .833).

A first preliminary coding served to exclude participants and trials for which the experimental protocol had not been followed correctly, or if parents had interfered in a relevant way in the procedure. For the remaining participants/trials, the children’s answers were transcribed by two different coders unfamiliar with the hypotheses of the study. Children’s answers to three test questions were coded: (1) the contents question, (2) the open source question, (3) the closed sources question.

Responses to the contents question were coded as correct when they mentioned what was inside the container or when they referred to objects looking closely similar to the actual objects inside the container (e.g., “a bus” for a tram car).

Responses to the open source question were coded for two measures. First, we generated a binary variable coding for whether children mentioned a source (irrespective of correctness) in response to the open source question (0 = no source mentioned; 1 = source mentioned). Second, we created a binary variable coding for whether children referred to the correct source in a given trial (0 = incorrect response; 1 = correct response). The first measure quantified the children’s willingness to provide a relevant response, while the second measure quantified the accuracy of this response. Coders were instructed to code any reference to visual perception (e.g., “I looked”, “I peeked” etc.) in response to the question as a reference to having seen the contents of the container. Conversely, any reference to the experimenter (such as pointing or addressing her) or to testimony (e.g., “someone told me”) was coded as a reference to having been told about the contents of the container. Finally, a lack of response after the question had been repeated once, an answer not referring to either perception, the experimenter, or testimony (such as “I just know”), as well as professions of ignorance (such as “I don’t know”), were coded as incorrect answers.

Responses to the closed source question were coded in a similar way as the responses to the open source question. However, for this test question, we did not code separately whether children mentioned a source but only whether their response was correct.

Responses of children in the final sample were excluded from analysis if the experimenter failed to repeat the question in case the child had not given a response to the initial query (Experiment 1: 6 responses; Experiment 2: 11 responses; Experiment 3: 14 responses).

A trial would also have been excluded from analysis if the children had not answered the contents question correctly. Since all children in all trials were able to provide a correct response to the contents question, no trials had to be excluded in this way. Trials were also excluded when either experimenter significantly departed from the experimental protocol, when the child did not cooperate, or when a caregiver interfered with the procedure—e.g., by giving hints to the child (Experiment 1: 2 trials; Experiment 2: 0 trials; Experiment 3: 2 trials). Post hoc analyses indicated that the number of excluded responses did not significantly differ between conditions.

## Results

### Non-parametric analysis

For a summary of the descriptive results for Experiments 1, 2, and 3, see Tables 1 and 2 in S3 File. As preregistered, we first carried out non-parametric analyses based on Wilcoxon Signed-Rank tests (see Table 1 for detailed statistics). These analyses were carried out on the proportions of answers in a given condition. All reported within-subject statistical comparisons were performed by two-tailed Wilcoxon Signed Ranks tests.

**Table 1:** Results of non-parametric analysis for Experiments 1, 2 (4-year-olds), and 3 (3-year-olds).

Experiment	Open Source Question								Closed Source Question			
	Sources Mentioned				Accuracy				Accuracy			
	Agreement		Source		Agreement		Source		Agreement		Source	
	*Z*	*p*	*Z*	*p*	*Z*	*p*	*Z*	*p*	*Z*	*p*	*Z*	*p*
Experiment 1	0.47	.635	0.17	.86	**1.92**	**.055**	1.63	.102	0.77	.440	0.49	.622
Experiment 2	0.18	.851	0.486	.627	**2.2**	**.028**	**2.84**	**.004**	0.50	.613	**2.37**	**.018**
Experiment 3	0.16	.873	0.14	.891	1.59	.111	**1.84**	**.065**	0.48	.627	1.11	.268

#### Number of sources mentioned

On average, children mentioned a source in response to the open source memory question (“How do you know that…?”) in 82.45% (SD = 24.87) of trials in Experiment 1, in 79.46% (SD = 32.24) of trials in Experiment 2, and in 40.69% (SD = 41.13) of trials in Experiment 3. In each of our experiments, there was no effect of Agreement or of Source on how many sources children mentioned in response to the open source memory question (all *p*s > .626, see Table 1). That is, across all three experiments, children mentioned a roughly equal number of sources in the agreement and the disagreement conditions as well as in the ‘seen’ and ‘told’ conditions.

#### Performance in the open source memory question

**Fig 1:**
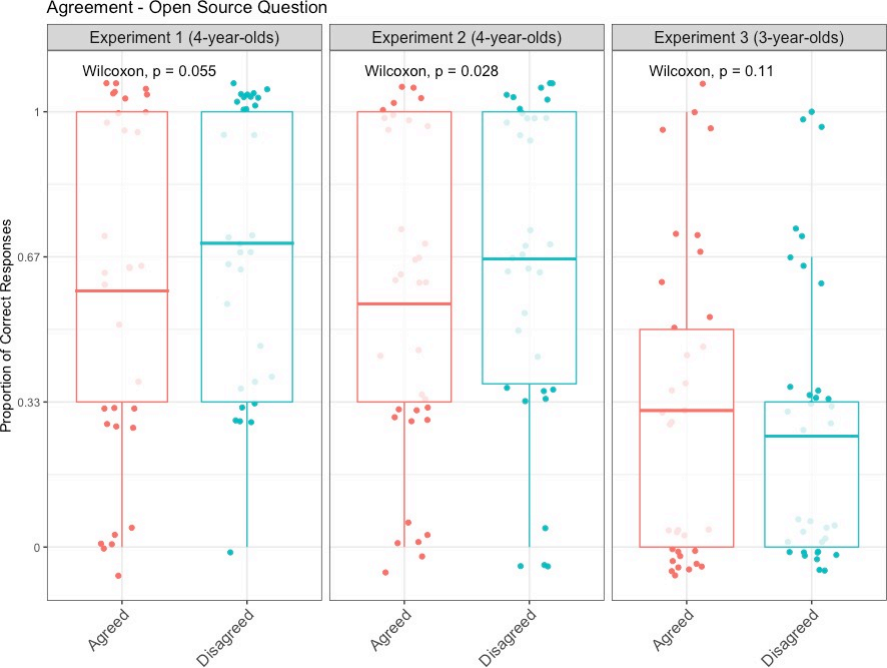
Results of the non-parametric analysis for the effect of agreement on children’s performance in the open source memory question. Box-plot middle lines depict means; scatter plots depict individual participant’s jittered proportions of correct responses. Experiments 1 and 2 were conducted with 4-year-olds, Experiment 3 with 3-year-olds.

On average, children answered the open source memory question correctly in 64.11% (SD = 31.07) of trials in Experiment 1, in 60.78% (SD = 33.15) of trials in Experiment 2, and in 28% (SD = 31.59) of trials in Experiment 3. Importantly, we found a marginally significant effect of Agreement on children’s responses to the open source question in Experiment 1 (*p* = .055), and a significant effect in Experiment 2 (*p* = .028; see Fig 1). We computed the combined probability for these two independent studies using the weighted Z-method [47]—weighting each study by its sample size. This analysis yielded a significant result (*p =* .005). This suggests that 4-year-old children in Experiment 2 (M_Disagreed_ = 0.66, SD_Disagreed_ = 0.48; M_Agreed_ = 0.56, SD_Agreed_ = 0.5), and to some extent in Experiment 1 (M_Disagreed_ = 0.69, SD_Disagreed_ = 0.46; M_Agreed_ = 0.59, SD_Agreed_ = 0.49), tended to perform better at recalling how they came to know about the contents of the container in the disagreement compared to the agreement condition. In contrast, 3-year-old children (Experiment 3) performed roughly equally in responding to the open source question when the puppet had disagreed (M_Disagreed_ = 0.27, SD_Disagreed_ = 0.45) and agreed with them (M_Agreed_ = 0.32, SD_Agreed_ = 0.47; *p* = .111).

Further, Source had a significant effect on children’s performance in the open source memory question in Experiment 2 (M_Told_ = 0.72, SD_Told_ = 0.45; M_Seen_ = 0.50, SD_Seen_ = 0.50, *p* = .004) and Experiment 3 (M_Told_ = 0.38, SD_Told_ = 0.41; M_Seen_ = 0.21, SD_Seen_ = 0.49, *p* = .065) such that children tended to perform better at recalling the correct source when they had been told about the contents of the container than when they had seen them.

#### Performance in the closed source memory question

On average, children answered the closed source memory question (“Did you see or were you told that…?”) correctly in 79.9% (SD = 21.24) of trials in Experiment 1, in 78.92% (SD = 20.79) of trials in Experiment 2, and in 52.79% (SD = 29.28) of trials in Experiment 3. Similarly to the open source question, children in Experiment 2 performed better at answering the closed source question when they had been told about the contents of the container (M_Told_ = 0.89, SD_Told_ = 0.31) compared to when they had seen them (M_Seen_ = 0.7, SD_Seen_ = 0.46, *p* = .018). Source and Agreement had no other effect on performance in the closed source memory question in any of the three experiments (all *p*s > .267, see Table 1).

### Mixed-effects analysis

To explicitly take into account repeated-measures, we entered the data of all three experiments (N = 100) into separate logistic linear mixed-effects models with a binomial distribution (logit link) for each of our dependent measures. That is, even though we report the results of these models together below, we ran three separate mixed-effect models, one for each of the following: (1) whether participants mentioned a source in response to the open source question, (2) whether participants provided the correct source in response to the open source question, and (3) whether participants answered the closed source question correctly.

This analysis allowed us to ask two questions. First, whether the effect of Agreement on children’s performance in the open source memory question increased with age. In other words, we looked for evidence that–with increasing age–children’s performance in the open source memory question became increasingly sensitive to whether their interlocutor had agreed or disagreed with them. If the effect of agreement on children’s source memory performance depended on age, we would expect to find a significant interaction effect between Age and Agreement here. Second, this analysis enabled us to test for interaction effects between the Source and Agreement factors. After all, it might be the case that the effect of disagreement depended on what source the interlocutor purported to have or vice versa.

We added random intercepts for each participant with fixed slopes as well as fixed effects for *Age* (in months), *Agreement (agreed/disagreed)*, *Source (seen/told)*, *Trial Number* (to control for order effects), *Agreement x Source*, *Age x Agreement*, and *Age* x *Source*. By-participant slopes for the various effects were not included because models including such slopes resulted in a singular fit. P-values were obtained by likelihood ratio tests (LRTs) of the full model with the effect in question against the model without the effect in question. For the estimates of the model parameters, the 95% confidence intervals were assessed by computing a likelihood profile and finding the appropriate cutoffs based on the LRT. Non-significant interaction effects were dropped from the model to interpret main effects. To assess main effects in the presence of a higher-order interaction (as was the case for the effects of Age and Agreement for children’s accuracy in the open source question), an LRT was conducted between models differing only in the presence or absence of the fixed main effect. Statistics were computed using R 3.6.2 (R Core Team, 2019) with the *lme4* package (*v1*.*1–21*; [48]). The results of this analysis are summarized in Table 2.

**Table 2:** Results of likelihood ratio test-based model comparison for children’s performance in Experiments 1, 2, and 3.

Dependent Variable	Model	*Df*	χ^2^	*p*	*95% CI*
Open Source Question: Sources Mentioned	**Trial Number**	**1**	**46.49**	**< .0001**	**0.44–0.88**
	**Age in months**	**1**	**32.36**	**< .0001**	**1.49–3.56**
	Agreement (agreed/disagreed)	1	0.001	.974	-0.62–0.6
	Source (seen/told)	1	0.30	.583	-0.43–0.77
Open Source Question: Accuracy	**Trial Number**	**1**	**14.96**	**< .001**	**0.13–0.41**
	**Age in months**	**1**	**16.42**	**< .0001**	**0.53–1.59**
	Agreement (agreed/disagreed)	1	2.04	.153	-0.13–0.80
	**Source (seen/told)**	**1**	**28.36**	**< .0001**	**-1.75 –-0.78**
	Age*Agreement	**1**	**5.67**	**.017**	**0.11–1.11**
Closed Source Question: Accuracy	**Trial Number**	**1**	**4.79**	**.029**	**0.01–0.26**
	**Age in months**	**1**	**24.17**	**< .0001**	**0.46–1.06**
	Agreement (agreed/disagreed)	1	0.51	.477	-0.57–0.26
	**Source (seen/told)**	**1**	**14.22**	**< .001**	**-1.23 –-0.38**

‘*’ denotes an interaction term in the model. The interaction between Age and Agreement on children’s performance in the open source question was the only significant interaction effect we found. All other, non-significant interaction terms were dropped from the model in order to interpret main effects. Significant effects at the .05 level are printed in bold.

#### Order and age effects

We found significant effects of Trial Number for all of our measures. This suggests that there were trial order effects in how many sources children mentioned in the open source question, as well as how often children were correct in responding to both the open and closed source questions.

However, in spite of these order effects, our mixed-effects analysis showed a main effect of Age for all measures: with increasing age, children mentioned more sources, and were more likely to correctly answer the open source memory question, as well as the closed source memory question.

#### Agreement effects

**Fig 2:**
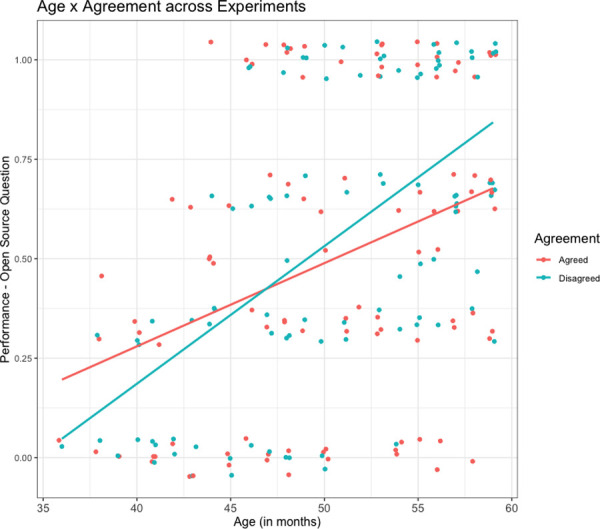
Relationship between age (in months) and agreement across all three experiments (N = 100). Scatter plots depict individual participants’ jittered performance (proportion correct) in the agreement and disagreement conditions. Regression lines depict the results of linear logistic regression for the effect of age on performance in the two Agreement conditions.

Crucially, we did not find main effects of Agreement, observing instead a significant interaction effect between Age and Agreement on performance in the open source memory question (see Fig 2). This suggests that, across all the experiments (and in spite of the order effects mentioned above), with increasing age children performed better in answering the open source question in the face of disagreement compared to agreement.

#### Source effects

We found a main effect of Source on performance both in the open and the closed source memory questions such that, independently of their age, children were more likely to provide the correct source when they had been told about the contents of the container. We did not find any interaction effects between Source and Agreement on any of our measures suggesting that the effect of Source did not depend on whether the interlocutor had agreed with the child.

## Discussion

### The effect of agreement

In the present study, 3- and 4-year-old children were asked by a puppet how they knew about the contents of a box, which they had either seen or been told about. The results of Experiments 1 and 2 indicate that 4-year-old children performed better in answering an open-ended source question when the puppet had disagreed rather than agreed with them about the contents of the box. In contrast, 3-year-old children in Experiment 3 did not show this effect.

Taken together Experiments 1 and 2 provided evidence for four-year-old children’s tendency to report sources more accurately when their interlocutor disagreed with them. Nonetheless, the current study had limitations that might explain why we did not observe a stronger effect of disagreement on children’s responses. Most importantly, children engaged in a ‘game’ with a puppet rather than in a ‘real-world’ interaction with more important communicative stakes. Furthermore, our agreement/disagreement manipulation was relatively subtle in so far as the only relevant difference between these conditions was one sentence spoken by the interlocutor puppet.

Another limitation is that our results do not reveal whether the effect of Agreement on 4-year-olds’ source memory is due to (i) an increase in performance in the disagreement condition, (ii) a decrease in performance in the agreement condition, or (iii) a combination of these two factors. In spite of these limitations and regardless of the respective effects of agreement and disagreement, our results nonetheless indicate that source memory performance was comparatively enhanced when children needed to justify their claims (in the disagreement condition) compared to when they did not (in the agreement condition), thus supporting the view that source memory can serve a social function in childhood.

As mentioned above, both the individual and social functions of source monitoring should be more strongly activated in the context of interpersonal disagreement. As a result, source memory performance should improve in such a context. However, the effect of Agreement on 4-year-olds’ source memory performance can be explained by a number of non-mutually exclusive mechanisms. First, disagreement (more so than agreement) might have caused children to doubt the truth of their own assertions thereby prompting them to search for internal justification.

Second, the interlocutor’s disagreement might have made it easier than agreement for children to understand what kind of response would be appropriate to the open source memory question via cueing children to interpret the question as a request for justification. As a consequence, in the context of disagreement, the open source question might have been interpreted as a retrieval cue for the events at the beginning of the trial (when children had found out about the contents of the container). Thus, it might have been easier for children to think of the event during which they found out about the contents of the container *as a source* of their belief [8]. In contrast, it might have been pragmatically less clear to children how to interpret the open source question in the context of agreement (for example, it might have been perceived as a ‘test question,’ [46]). On this interpretation, our results suggest that 4-year-olds understand the justificatory function of source information.

Third, agreement might have impacted children’s motivation to either convince their interlocutor or hedge their bets about their own claim. Children might have been prompted to be more careful in answering correctly to a disagreeing (i.e., epistemically vigilant) interlocutor than to an agreeing one. As a result, the participants might have spent more cognitive resources on recovering the exact source of their belief about the contents of the box in the context of disagreement. In other words, children had stronger incentives to calibrate their commitment correctly when facing a disagreeing rather than an agreeing interlocutor. The fact that children made more source errors in the agreement than the disagreement condition despite the fact that they did not mention fewer sources, lends support to this interpretation.

Note that this latter interpretation of the results attributes a higher degree of communicative competence to children. The 4-year-old children in Experiments 1 & 2 would not only have to have understood that questions in the context of disagreement can be interpreted as requests for justification and that sources can provide such justification: they would also have to have understood that sources can serve the more specific function of modulating speaker commitment.

It is also worth noting that, since in any given trial the children did not know whether their interlocutor would agree or disagree with them, the effect of Agreement on 4-year-olds’ performance has to be attributed to the retrieval, and not to the encoding, of source information. One might think that our data could be explained by the fact that children might have come to expect to encounter disagreement in subsequent trials after the first ‘disagreement trial,’ and encoded source information more deeply as a result. Such a generalized expectation should, however, have resulted in increased performance across trials, and not only in disagreement trials. Moreover, since we controlled for the effect of Trial Number in our models, it is unlikely that the effect we observed was driven by children who received a disagreement trial first or by inter-trial carry-over effects of another sort. Moreover, an effect driven by the retrieval phase is to be expected under the assumption that source monitoring processes specifically occur at retrieval and operate over episodic representations [10]. In short, regardless of the exact mechanisms leading to the effect of disagreement on source recall, the present results therefore support the idea that retrieval of source information is sensitive to communicative context by four years of age [31].

We make no strong claims about why Agreement had no effect on the performance of three-year-olds. Their insensitivity to Agreement in our task can be explained by a variety of factors, including a reduced sensitivity to disagreement, weak source memory capacities, or linguistic difficulties. Yet, the effect of communicative context on source memory performance observed here might contribute to our understanding of the underlying reasons for the common finding that source memory performance significantly increases between three and five years of age. In the present study, older children did not only perform better but also became sensitive to agreement with their interlocutor in their responses to the open source memory question.

The improvement in source memory performance between three and five years is often attributed to children’s developing theory-of-mind abilities (e.g., [13, 49, 50]). However, while it is possible that source memory performance increases as a function of proficiency in mental state reasoning, another contributing factor might be that children’s increasing source memory performance is related to their developing communicative competence. In particular, it has been shown that children develop a range of important communicative capacities related to epistemic vigilance during this time (e.g., [23]). Similarly, we observed an increased effect of disagreement on children’s source memory performance as they got older. Importantly, this effect of disagreement cannot be explained merely as a result of a generalized increased proficiency in source memory with age (since the effect is specific to the disagreement condition). Instead, it could be the result of the maturation of communicative competence (e.g., an increased understanding that disagreement implies a request for justification). Our results thus add to this prior work by suggesting that 4-year-olds might be sensitive to the communicative function of knowing the source of their own beliefs as providing a potential justification of their assertions to their interlocutor.

### The effect of source

In both age groups, the children were more likely to accurately report that they had been told something than that they had seen something. Although we did not predict this result, it deserves discussion given that it contrasts with other findings suggesting that children have a tendency to over-attribute their own beliefs to perception rather than testimony or inference. In particular, Whitcombe and Robinson (2000) [21], as well as Robinson and Whitcombe (2003) [22], found a trend opposite to that of the present study: in their studies, children of the same age group seemed to be biased to over-report first-hand over second-hand sources of information (for similar results with adults, see [51]).

In our view, this result can be explained in several ways. On the one hand, differences in the way the information was presented between different source conditions in the present study might have made it easier for the children in our experiment to encode that they had been told the answer, or more difficult to encode that they had seen the contents of the container. In particular, ‘seeing’ something may not have been encoded as a unique event while the interlocutor’s communicative act might have made event boundaries easier to differentiate. However, the differences between the protocols of the current and of past studies in this respect were not large, and so it is not clear why such differences at encoding would emerge.

On the other hand, this effect could have been due to a response bias towards answering having been told, independently of the actual source. That is, children might have been better at reporting having been told because they were choosing this answer more often overall and not because they were actually better at remembering having been told. One reason for such a response bias in the present study might be that the children either wanted to hedge their bets or increase their authority by referring to the experimenter as a source. This possibility is supported by results suggesting that preschool children generally view testimony as highly trustworthy [52, 53]. In the current context, results such as those by Jaswal and colleagues suggest that children perceived the adult informant as authoritative, and might therefore have judged it as ‘safer’ to refer to testimony when asked for their source. The main difference between our current design and previous studies such as those by Whitcombe and Robinson is that source memory questions were asked in a naturalistic conversational context by a ‘second party’ interlocutor rather than in a test situation by a ‘third party’ experimenter. This might have biased children to want to manage conversational risk in their answers. If that was the case, it would further support the idea that children of this age can recognize how source report modulates speaker commitment. Alternatively, a response bias might have been caused by the fact that many of the children’s beliefs do in fact come from testimony. Children might therefore have reported having been told whenever they were unsure about their actual source because they have a baseline assumption that any given piece of knowledge they have is likely to come from others.

## Conclusion

The current study suggests that by four years of age, children’s source memory retrieval is sensitive to communicative context. This sensitivity likely forms the basis of an understanding of the costs and benefits of source report for the purposes of hedging one’s bets or claiming authority. Future studies should thus aim to disentangle whether children of this age group are already sensitive to the communicative costs and benefits of different source reports.

In addition, we also found that, in contrast to earlier studies, children performed better at reporting their source when their belief was based on testimony than when it was based on perception. This effect might have been due to a bias on the children’s part to over-report testimony as the source of their belief. Such a bias could be explained either by children’s attempt to manage conversational risk or by a baseline assumption about the origins of their knowledge in social sources.

## Supporting information

S1 File Counterbalancing orders and assignments for Experiments 1, 2, and 3. (DOCX)

S2 File Full protocol for Experiments 1, 2, and 3. (DOCX)

S3 File Mean and standard deviations for all dependent measures in the two agreement (Table 1) and source (Table 2) conditions in Experiments 1, 2, and 3. (DOCX)
