# Supplementary material for: The effect of disagreement on children’s source memory performance
Source: PLoS One. 2021 Apr 9;16(4):e0249958. doi: 10.1371/journal.pone.0249958 (PMC8034710; doi:10.1371/journal.pone.0249958)
Supplement: S1 File — (DOCX) [file pone.0249958.s001.docx]

**S1 File.**

**Counterbalancing orders**

**Table 1:** Counterbalancing orders for Experiments 1, 2, and 3.

**
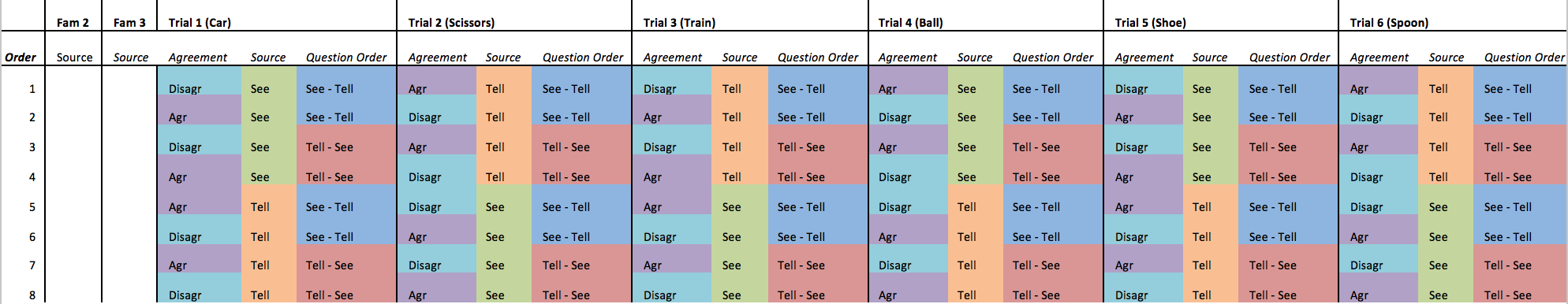
**

**Counterbalancing assignments for each experiment**

**Table 2**: Counterbalancing assignments for Experiments 1, 2, and 3.

|  | | Order | | | | | | | |
| --- | --- | --- | --- | --- | --- | --- | --- | --- | --- |
|  |  | 1 | 2 | 3 | 4 | 5 | 6 | 7 | 8 |
| Number of children | Experiment 1 – 4-year-olds | 4 | 4 | 4 | 4 | 4 | 4 | 4 | 4 |
|  | Experiment 2 – 4-year-olds | 4 | 2 | 5 | 5 | 5 | 5 | 5 | 3 |
|  | Experiment 3 – 3-year-olds | 3 | 5 | 5 | 5 | 5 | 3 | 4 | 4 |
