# Supplementary material for: The effect of disagreement on children’s source memory performance
Source: PLoS One. 2021 Apr 9;16(4):e0249958. doi: 10.1371/journal.pone.0249958 (PMC8034710; doi:10.1371/journal.pone.0249958)
Supplement: S2 File — (DOCX) [file pone.0249958.s002.docx]

**S2 File.**

**Object/box contents:**

***Familiarization trials***

Fam 1 – Pen

Fam 2 – Book

Fam 3 – Phone

***Test Trials***

Test 1 – Car

Test 2 – Cup

Test 3 – Train

Test 4 – Ball

Test 5 – Shoe

Test 6 – Spoon

***“Disagreement objects” (objects proposed as alternatives by Zirmi):***

1^st^ Disagreement Trial: Watch

2^nd^ Disagreement Trial: Apple

3^rd^ Disagreement Trial: Glasses

**Study Protocol**

***Familiarization 1***

*Experimenter introduces the child to ‘Zirmi” the cat (interlocutor puppet controlled by Experimenter 2)*

Experimenter: “Hello! This is Zirmi. We will play a game together. You will have to guess what’s inside this box! Do you want to do that?”

*Experimenter* *then brings box 1 (containing object 1) out from behind the curtain*

Zirmi: “Oh! Look at this nice box! What do you think is inside?”

*Child answers*

Zirmi: “Should we look inside to find out what’s inside?”

*Zirmi takes the box and opens it so that the child can see*

Zirmi: “Wow! It’s a pen! There is a pen inside!”

***Familiarization 2***

*Experimenter takes the object visibly out of the box and puts a new object inside behind the curtain. Experimenter puts the box back on the table leaving her hand on it.*

[See source]

“Do you want to look inside to find out what’s inside?”

*After the child has opened the box and looked inside*

“Did you see what’s inside? Can you tell me?”

*In case the child does not give the correct answer, the experimenter asks the child to look inside again until the child gives the right answer.*

*After the child told the experimenter*

“Wow! That’s exciting!”

“How did you find out what was inside?”

(*If child answers incorrectly or doesn’t answer, the experimenter corrects the child*)

*Zirmi appears and sits on the box. Experimenter 1 turns away.*

Zirmi: “Oh! Look there is the box again. What do you think is inside this time?”

*Child answers*

Zirmi: “Hm… I think there is a bottle inside. Let’s look what’s inside”

*Zirmi takes the box and opens it so that the child can see.*

Zirmi: “Wow! It’s a book! There is a book inside! I was wrong…”

***Familiarization 3***

*Experimenter takes the object visibly out of the box and puts a new object inside behind the curtain. Experimenter puts the box back on the table leaving her hand on it until Zirmi appears.*

Experimenter: “Look I put something new inside! Do you want to know what it is?“

[Tell source]

Experimenter: “I will tell you! It’s a phone! There is a phone inside this box!”

“What’s inside? Can you tell me?”

“How did you find out what was inside?”

(*If child answers incorrectly or doesn’t answer, the experimenter corrects the child*)

*Zirmi appears and sits on the box. Experimenter 1 turns away.*

Zirmi: “Oh! Look there is the box again. What do you think is inside this time?”

*Child answers (repeat the question until the child says “phone”)*

Zirmi: “Hm… I think you are right… there is a phone inside.”

*Zirmi takes the box and opens it so that the child can see.*

Zirmi: “Yes! It’s a phone!”

***Transition***

Zirmi: “Oh! I have to go…!”

***Test***

*Experimenter takes the object visibly out of the box and puts a new object inside behind the curtain. She then puts the box back on the table leaving her hand on it.*

Experimenter: “Look I put something new inside! Do you want to know what it is?“

[Tell condition]

Experimenter: “I will tell you! It’s an X! There is an X inside this box!”

“What’s inside? Can you tell me?”

[See condition]

“Do you want to look inside to find out what’s inside?”

“Did you see what’s inside? Can you tell me?”

*In case the child does not give the correct answer, the experimenter asks the child to look inside again until the child gives the right answer.*

*After the child told the experimenter*

“Wow! That’s exciting!”

*Zirmi appears and sits on the box. Experimenter 1 turns away.*

Zirmi: “Hello! Oh! There is the box again… What do you think is inside?”

*After child answers*

[Agreement condition]

Zirmi: “You are right! There must be an X inside.”

[Disagreement condition]

Zirmi: “No! I don’t think there is an X inside! I think there is a [disagreement object] inside!”

[Source question 1]

Zirmi: “How do you know there is an X inside?”

*After child answers*

[Source question 2 – order 1]

Zirmi: “Did you see what’s inside or did someone tell you?”

[Source question 2 – order 2]

Zirmi: “Did someone tell you what’s inside or did you see it?”

[Disagreement condition]

Zirmi: “Hm.. if you [saw/were told] then you must be right… there must be an X inside.”

Zirmi: “Oh! I have to go…!.”

[Zirmi leaves]
