# Supplementary material for: The effect of disagreement on children’s source memory performance
Source: PLoS One. 2021 Apr 9;16(4):e0249958. doi: 10.1371/journal.pone.0249958 (PMC8034710; doi:10.1371/journal.pone.0249958)
Supplement: S3 File — (DOCX) [file pone.0249958.s003.docx]

**S3 File.**

**Descriptives**

**Table 1**: Descriptive results for all three dependent measures in each Agreement condition for Experiments 1, 2, and 3.

| Experiment | Agreement | Open Source Question | | | | Closed Source Question | |
| --- | --- | --- | --- | --- | --- | --- | --- |
|  |  | Sources Mentioned | | Accuracy | | Accuracy | |
|  |  | M | SD | M | SD | M | SD |
| Experiment 1 | Interlocutor agreed | 0.81 | 0.39 | 0.59 | 0.49 | 0.76 | 0.43 |
|  | Interlocutor disagreed | 0.83 | 0.37 | 0.69 | 0.46 | 0.83 | 0.38 |
| Experiment 2 | Interlocutor agreed | 0.78 | 0.41 | 0.56 | 0.5 | 0.81 | 0.4 |
|  | Interlocutor disagreed | 0.79 | 0.41 | 0.66 | 0.48 | 0.78 | 0.42 |
| Experiment 3 | Interlocutor agreed | 0.41 | 0.5 | 0.32 | 0.47 | 0.54 | 0.50 |
|  | Interlocutor disagreed | 0.43 | 0.5 | 0.27 | 0.45 | 0.54 | 0.50 |

**Table 2:** Descriptive results for all three dependent measures in each Source condition for Experiments 1, 2, and 3.

| Experiment | Source | Open Source Question | | | | Closed Source Question | |
| --- | --- | --- | --- | --- | --- | --- | --- |
|  |  | Sources Mentioned | | Accuracy | | Accuracy | |
|  |  | M | SD | M | SD | M | SD |
| Experiment 1 | Seen contents | 0.83 | 0.38 | 0.58 | 0.5 | 0.76 | 0.43 |
|  | Told about contents | 0.83 | 0.38 | 0.72 | 0.45 | 0.83 | 0.38 |
| Experiment 2 | Seen contents | 0.81 | 0.4 | 0.50 | 0.50 | 0.7 | 0.46 |
|  | Told about contents | 0.77 | 0.42 | 0.72 | 0.45 | 0.89 | 0.31 |
| Experiment 3 | Seen contents | 0.43 | 0.5 | 0.21 | 0.41 | 0.48 | 0.50 |
|  | Told about contents | 0.42 | 0.5 | 0.38 | 0.49 | 0.59 | 0.49 |
